# Process and effect evaluation of a referral aid for smoking cessation counselling in primary care: Findings of a randomized controlled trial

**DOI:** 10.18332/tpc/176148

**Published:** 2024-01-10

**Authors:** Daniëlle N. Zijlstra, Catherine A. Bolman, Jean W. Muris, Hein de Vries

**Affiliations:** 1 Department of Health Promotion, Maastricht University/CAPHRI, Maastricht, The Netherlands; 2 Department of Health Psychology, Open University of the Netherlands, Heerlen, The Netherlands; 3 Department of General Practice, Maastricht University/CAPHRI, Maastricht, The Netherlands

**Keywords:** smoking cessation, evidencebased interventions, general practice, primary care, nurse practitioners

## Abstract

**INTRODUCTION:**

This study explored the use and effects of a smoking cessation referral in: 1) practice nurses (PNs), and 2) smokers. The use of evidence-based smoking cessation interventions (EBSCIs) can double the likelihood of a successful smoking cessation attempt. A referral aid was developed to aid Dutch PNs in primary care in deciding which smokers are the most suitable for EBSCI.

**METHODS:**

Two different studies were conducted: 1) a randomized controlled trial with a process evaluation (n=82) and effect evaluation (n=285) among smoking patients recruited by PNs (n=73), and 2) a process evaluation among a subgroup of PNs (n=40) from January 2019 to September 2020.

**RESULTS:**

Overall, the response in both groups was low. PNs found the referral aid materials clear and understandable. Smokers had similar but (slightly) less-positive opinions. The smokers in both groups did not differ in the amount of discussion and use of EBSCIs, nor on smoking abstinence.

**CONCLUSIONS:**

Further research should assess how to better involve PNs and smokers when recruiting for an RCT and how to foster effective counselling. Additional research should also look deeper into barriers to referral of both PNs and smokers, and how to stimulate referral to EBSCIs best and help smokers to make a decision; for example by implementing a simplified strategy both within the primary care setting and outside, by involving other healthcare professionals or options outside healthcare such as the workplace and social domain.

**Trial registration:**

The study was registered at the Netherlands Trial Register (NL7020, https://www.trialregister.nl/trial/7020).

## INTRODUCTION

Smoking is responsible for 13% of Dutch morbidity, resulting in about 20000 deaths per year^1^ and causing a burden of around €33 million in healthcare costs, decreased work productivity, and premature death^2^. Consequently, several actions have been undertaken to support smoking cessation at the policy level (e.g. public smoking restrictions and regular tax increases)^3^, the organizational level (e.g. national smoking restrictions in workplaces and smoking cessation interventions specially targeted at organizations)^4^, and at the individual level, for instance via mass media campaigns^5^ or via healthcare such as the primary care settings (PCS)^6,7^, midwives^8^, nurses working on coronary wards^9^ or other healthcare professionals (HCPs)^10^. As most smokers visit their PCS at least yearly, the PCS can serve as a valuable access point for reaching smokers, stimulating them to quit and use evidence-based interventions^11^.

Within the Dutch PCS, most smoking cessation counselling is provided by a trained practice nurse (PN)^12^. In collaboration with the general practitioner, PNs provide smoking cessation counselling according to a structured, evidence-based counselling guideline^13^, which is similar to the 5As (Ask, Advise, Assess, Assist, and Arrange) strategy^14^. The Dutch guide has seven steps: 1) providing quit advice, 2) assessing a smoking profile, 3) assessing and increasing motivation, 4) exploring, discussing, and, when possible, removing existing barriers, 5) discussing cessation aids, 6) helping to set a quit date and developing a quit plan; and 7) offering support after the quit date.

Yet, these steps are not always used, in particular providing information on evidence-based smoking cessation interventions (EBSCIs)^15,16^, because PNs may have insufficient knowledge about them^16^. Using readily available EBSCIs such as face-to-face counselling, eHealth^17^, telephonic counselling^18^, group counselling^19^, nicotine replacement therapy (NRT), or pharmacotherapy^20^, can double the chance of a successful smoking cessation attempt^21^. Referring to EBSCIs may enable a PN to improve the quality of their work. It may help smokers to identify a method most suitable to their needs, resulting in more involvement and commitment of smokers in their own chosen cessation method and their cessation attempt^22^.

A referral aid was developed and evaluated to aid Dutch PNs and other healthcare providers in primary care in referring smokers to EBSCIs. This article describes the evaluation study and is divided into three parts: 1) the recruitment and retention of participants, 2) a process evaluation; and 3) an effect evaluation. During the study, the perspective of two user groups was taken into account, namely PNs (responsible for implementing the referral aid and recruiting smokers) and smokers (end users).

## METHOD

### Design and intervention

The referral aid was named the ‘StopWijzer’, which can be translated as both stop-indicator and stop-smarter. The study consisted of a multi-site, two-group, parallel-randomized controlled trial involving experimental and control conditions. The PNs in the control condition provided care as usual, by the seven steps from the Dutch treatment guideline for tobacco addiction and smoking cessation support^13^. The PNs in the experimental condition received an intervention manual to aid them in discussing smoking cessation with smokers and to help them select an EBSCI that fits the patient’s needs and preferences (extension on step 5 of the Dutch Cessation Guidelines). An overview of the different parts of the study is given in Table 1. In the first part of the study, the recruitment of PNs and smokers, and the retention of the smokers, are described. In the second part, the use and appreciation of the referral aid materials from both groups are evaluated (process evaluation). In the third part, the effect on: 1) use of EBSCIs, 2) decisional conflict, 3) quality of life, and 4) abstinence and smoking behavior of smokers is evaluated (effect evaluation). A full description of the referral aid and the design of the RCT can be found elsewhere^23^.

**Table 1:** Overview of the different parts of the RCT

*Part of the study*	*Sample*	*Sample size n*	*Objective*
Part 1: Recruitment and retention	PNs	73	Tracking the recruitment and adherence rate of PNs at the outset of the RCT
	Smokers	285	Tracking the recruitment and adherence rate of smokers at recruitment, baseline and at follow-up at 6 months
Part 2: Process evaluation	PNs (subsample)	40	Evaluating the use and appreciation of the referral aid materials by the PNs
	Smokers	82	Evaluating the use and appreciation of the referral aid materials by the PNs
Part 3: Effect evaluation	Smokers (same sample)	82	Measuring the effect on: 1) use of EBSCIs, 2) decisional conflict, 3) quality of life, and 4) abstinence and smoking behavior of smokers

The study proposal was approved by the medical ethics committee of the University Hospital Maastricht and Maastricht University (WMO, 2018-1038) and registered at the Netherlands Trial Register (NL7020, https://www.trialregister.nl/trial/7020).

*Referral aid*

**Figure 1:**
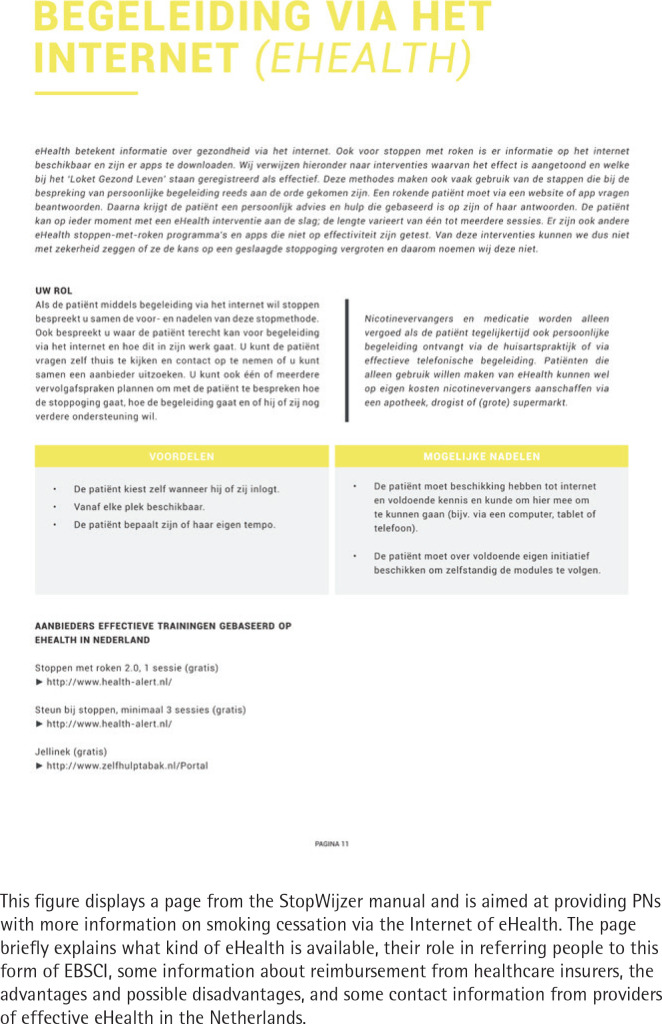
eHealth page

**Figure 2:**
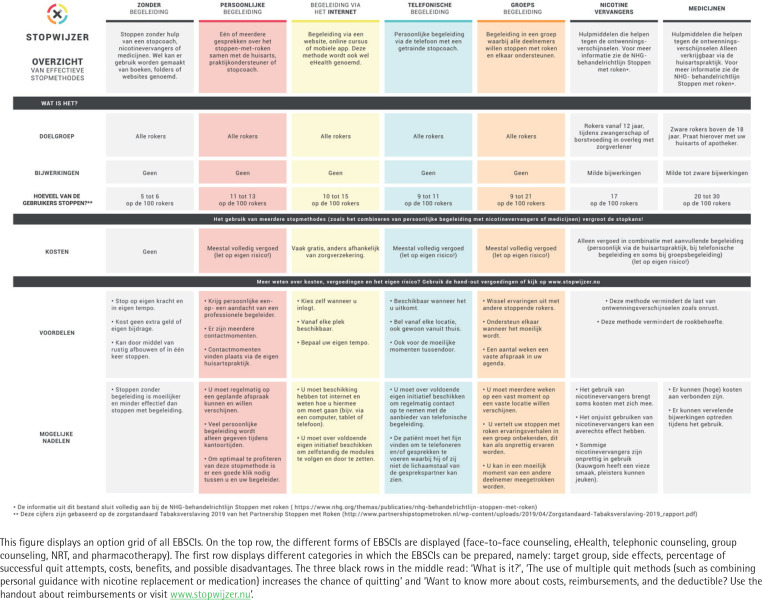
Option grid available EBSCIs

Materials concerning the referral aid were delivered to the PN as a small (letterbox-sized) package sent via post. The content of the referral aid was based on a needs assessment comprising a literature review^6,13,24,25^, individual semi-structured interviews among GPs (n=5), PNs (n=20) and smokers (n=9), a Delphi study on the referral to EBSCIs^16^ and the input of an advisory board consisting of experts representing various Dutch smoking cessation related organizations. The StopWijzer materials packages included the following items:

1. A manual (A4 size, approximately 20 pages), providing: a) an introduction and explanation of the aim of the referral aid; b) instructions on the use of the referral aid protocol, including a roadmap detailing the steps of the protocol and a flow-chart; c) an overview of reimbursement; d) an overview of the different readily available EBSCIs (face-to-face counseling, eHealth, telephonic counseling, group counseling, NRT and pharmacotherapy) including discouraging remarks on the use of non-EBSCIs (acupuncture, hypnotherapy, laser therapy and the use of e-cigarettes as a means to quit); e) a short guideline for follow-up consultations; and f) concluding remarks and room for taking notes (Figure 1).
2. A separate handout (A5 size, printed on both sides) containing a visualization of the most important concepts of the manual (the same flow-chart as in the manual), and a summary of the health insurers’ reimbursement policies.
3. An overview of the different EBSCIs (option grid or decision matrix; A3 size, laminated placemat), explaining the target groups, strengths and weaknesses, effectiveness, and costs of the mentioned EBSCIs (Figure 2).
4. Supplementary materials for the promotion of the study include information flyers aimed at informing smokers about the study, business cards, posters (paper and digital), and a pen and notebook featuring the logo of the referral aids.

All materials were written in clear and comprehensible language in accordance with the applicable Dutch guidelines (language level B1)^26^ and were also available on the referral aids’ website (only accessible for the experimental condition). This website also included a frequently asked questions (FAQ) page tailored to both conditions.

PNs in the experimental condition were asked via the information letter to read the manual at the start of the study to inform themselves of the information regarding the EBSCIs. Other materials, e.g. the option grid, could be implemented during counselling sessions in a way PNs saw fit. The PNs were encouraged in the manual to provide flyers and other materials to smokers and direct them to the study’s website, where smokers in the experimental group could also access all information from the materials. No formal training was provided to use the materials, but PNs were able to ask the research team questions if necessary.

### Procedure of the study

*Part 1: Recruitment and retention of participants (smokers and PNs)*

**Figure 3:**
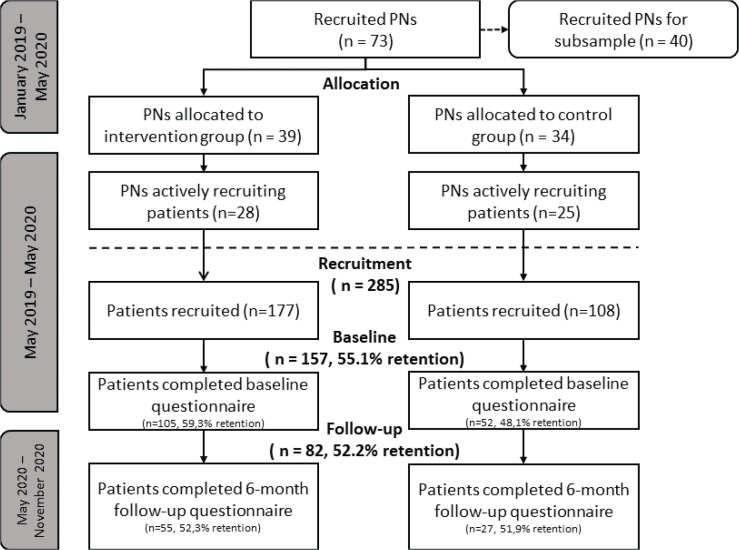
Recruitment process during the research

PCS were approached during January 2019 until May 2020 to recruit PNs to take part in the RCT (see Figure 3 for all time periods). PNs were recruited to: 1) recruit smokers, and 2) in the case of the experimental condition, refer smokers to EBSCIs in accordance with the method described in the referral aid. PNs were eligible if employed by at least one general practice in the Netherlands and providing smoking cessation counseling at least once a week.

A study invitation letter and a summary of the referral aids aim were sent to Dutch PNs using three approaches: 1) to three Dutch primary care associations (PCAs) in the south of the Netherlands collaborating with Maastricht University; 2) to individual PCS in the rest of the Netherlands via post and, when publicly known, via email (two weeks after the initial recruitment message, a minimum of three attempts via telephone per PN were made to achieve a more active and personal form of recruitment); and 3) via national congresses and advertisements in trade magazines or websites of relevant organizations, e.g. the Dutch ‘Quality register for smoking cessation (*kwaliteitsregister stoppen-met-roken,* www.kabiz.nl )’.

PNs expressing interest in participating were sent a more detailed information letter for the study and were asked to sign a study participation form. PNs were randomized in a 1:1 ratio on the practice level, in order of registration. As PNs from the experimental condition were provided with the referral aid and PNs from the control condition were only asked to provide care as usual (no additional intervention), blinding of the PNs was impossible.

Participating PNs were each requested to recruit 10 to 20 smokers [based on the sample size calculation of 292 patients with an effect size (odds ratio) of 0.30, a power of 0.80, and an alpha of 0.05]^23^. To stimulate active recruitment and prevent attrition, PNs were offered remuneration based on the number of recruited smokers (up to €100 for recruiting >15 smokers). To facilitate recruitment of smokers, regular contact by phone was maintained with PNs who did not register smokers, to remind them of participation and to provide them with tips from other PNs to recruit smokers who are not motivated to participate in the study. In addition, PNs received several other reminders, such as personal postcards and monthly newsletters, which were tailored by name and number of recruited smokers. The newsletter included personal success stories and recruitment tips from other participants, as well as recruitment tips based on literature.

At the end of the recruitment period (September 2020), all participating PNs were invited via email to take part in a process to evaluate the course of the RCT. The email provided a link to an online questionnaire and a summary of the referral aid and associated materials. The questionnaire took 15 minutes to complete, excluding the time PNs from the control condition needed to review the materials. PNs who did not respond within seven days were sent a maximum of two reminders. On completion, PNs received a €20 gift voucher as reimbursement.

The recruitment of smokers for the RCT occurred from May 2019 until May 2020. PNs were requested to inquire about the smoking habits of all smokers they spoke to during their consultations. Inclusion criteria for smokers were: use of tobacco products, aged ≥18 years, and able to read and understand the Dutch language. Those who only used e-cigarettes were not eligible.

Smokers who were eligible and willing to participate in the study were registered by the PN and received an information letter on their participation. Then, they received smoking cessation counseling with or without referral advice, depending on the condition to which the PN was assigned. Written informed consent was obtained from all participants at the start of the baseline questionnaire. Smokers were semi-blinded, as they were unaware of the procedure of any other group than the one they attended. Smokers were recruited to fill in two questionnaires: one at baseline and one at follow-up at 6 months. Smokers who filled in both questionnaires were rewarded with a gift voucher of €10.

*Part 2: Process evaluation*

To measure the use and appreciation of the materials by both PNs as smokers, as well as the course of discussing the different EBSCIs, a process evaluation was conducted during and alongside the RCT (i.e. only in the smokers and PN of the experimental group in the RCT; more details in Supplementary file Table 1 and the protocol publication^23^).

*Part 3: Effect evaluation*

The goal of the effect evaluation was to determine the referral aid’s effect on: 1) use of EBSCIs, 2) decisional conflict, 3) quality of life, and 4) smoking abstinence and smoking behavior (more details in Supplementary file Table 1 and the protocol publication^23^).

### Data analysis

The qualitative data were analyzed using input from the open questions belonging to the process evaluation, which were summarized in the text. For parts 1 and 2, differences in the reporting of use of the materials were analyzed using Person’s chi-squared tests on data of PNs from the subsample, and smokers who quit smoking after the intervention and those who did not. Appreciation of the materials were analyzed using independent sample t-tests to test for differences between the same groups of PNs and smokers.

For the RCT, descriptive analyses were conducted to describe the sample characteristics. Dropout analysis using chi-squared tests and t-tests were used to detect differences between smokers retained at the follow-up at 6 months and those who dropped out. Pearson’s chi-squared tests were used to compare intervention effects on the discussion of EBSCIs according to PNs and the actual usage of EBSCIs by smokers. Differences between conditions on 24-hour point prevalence abstinence, 7-day point prevalence abstinence, and 6-month prolonged abstinence, were assessed using Pearson’s chi-squared test on complete cases and negative scenarios (intention-to-treat principle)^27^. Initially, depending on the progress of the recruitment, a cost-effectiveness analysis (CEA) and a cost-utility analysis (CUA) were also planned to be conducted^23^.

## RESULTS

### Part 1: Recruitment and retention

*Practice nurses*

A total of 1663 PCS were approached to take part in the RCT of which 73 took part (4.4%). First, the recruitment of practices via the three participating PCAs resulted in 19 PNs out of 420 PNs associated with the PCAs (4.5%). Second, 1243 PCS that were not part of these PCAs were individually contacted. This resulted in 54 PNs (4.3%) willing to participate in the RCT. Attempts to contact potential participating PNs were sometimes cut off by the practice operator or assistant (the reasons provided included a demanding workload, upcoming employee leave, and previous or current participation in other studies). PNs who were reached but did not want to participate explained that they did not have the time, were on special leave within the RCT-period, or had recently moved or would move practice.

Third, the referral aid was promoted at two national congresses and via advertisements in trade magazines issued by the participating universities or smoking cessation associations. This did not yield any PCS or PNs willing to participate.

*Participating smokers*

From May 2019 till the end of May 2020, the 73 participating PNs recruited 285 smokers to take part in the RCT. Although PNs were each asked to recruit at least 10 smokers, recruitment rates varied widely between PNs. A total of 20 PNs did not recruit a single patient (n=11 in the experiment condition and n=9 in the control condition). Of the PNs that did recruit patients, PNs in the experimental condition (N=28) recruited an average of 6.12 smokers (SD=4.9) in comparison with 5.04 smokers (SD=4.8) by 25 PNs in the control condition. This difference in the number of patients per PN was not significant.

Of the total 285 participants registered by the PNs, 157 participants filled in the baseline questionnaire, of which 105 were included by PNs in the experimental condition and 52 participants by PNs in the control condition (see Figure 3 for more details and retention rates). The recruitment rate, as well as the retention rate at 6 months, did not significantly differ between the experimental and control conditions.

### Part 2: Process evaluation

*Practice nurses*

Recruitment among PNs for participation in the process evaluation yielded 40 PNs: 22 in the intervention condition and 18 in the control condition (Table 2). The process evaluation showed that PNs from the experimental condition used the placemat to describe the different available EBSCIs and discuss the details of their advantages, disadvantages, costs, and use. The digital poster, which they could display on a screen in their waiting room, was used the least. These PNs reported a relatively high appreciation of the materials, resulting in a score of 8.8 (SD=0.9).

**Table 2:** Process evaluation among practice nurses (use and appreciation of the materials and intervention effects on the discussion of EBSCIs)

*Use of materials*	*PNs (N=22 from experimental condition) % (n)*				
Poster (paper)	68.2 (15)				
Poster (digital)	31.8 (7)				
Flyers	59.1 (13)				
Placemat	72.7 (16)				
Website during consultation	50.0 (11)				
Appreciation (I found the materials to be …)*	***PNs (N=22 from experimental condition) mean (SD)***				
Clear	4.14 (0.8)				
Understandable	4.23 (0.7)				
Educational	3.91 (0.6)				
Score (1–10)	8.68 (0.9)				
***Discussion of materials***	***Total (N=40) % (n)***	***Experimental condition (N=22) % (n)***	***Control condition (N=18) % (n)***	***χ^2^***	***p***
Counseling: GP-setting	100 (40)	100 (22)	100 (18)	-	-
Counseling: coach	25 (10)	36.4 (8)	11.1 (2)	3.37	0.067
EHealth	87.5 (35)	77.3 (17)	100.0 (18)	4.68	**0.031**
Group counseling	82.5 (21)	72.7 (16)	27.8 (5)	8.02	**0.005**
Telephone counseling	70 (28)	77.3 (17)	61.1 (11)	1.23	0.267
NRT	100 (40)	100 (22)	100 (18)	-	-
Pharmacotherapy	100 (40)	100 (22)	100 (18)	-	-
Other non-EBSCI	55 (22)	45.5 (10)	66.7 (12)	1.80	0.180

* 1= strongly disagree; 5=strongly agree.

All PNs indicated discussing possibilities for smoking cessation counseling in the GP setting, NRT, and pharmacotherapy. Counseling via an external smoking cessation coach was discussed the least among both PNs from the experimental and the control conditions. Conditions differed significantly on the rate of discussing eHealth (more often discussed in the control condition) and group counseling (more often discussed in the experimental condition). Other non-EBSCIs that PNs discussed included different variations of quitting such as quitting ‘cold turkey’, i.e. without quit-aids (n=21), acupuncture (n=17), laser therapy (n=9), and hypnosis (n=8). PNs indicated that although these options were discussed, this happened mostly at the request of the patient and without the endorsement of the PNs themselves.

*Participating smokers*

Flyers and paper posters were seen or received by more than half of the smokers in the experimental group. Around a quarter of all smokers indicated that they saw the digital poster in the waiting room, discussed the placemat during the consultation with their PN or visited the website during the consultation. Furthermore, smokers appreciated the materials [score of 8.0 (SD=1.8), range: 1–10]. Use and appreciation did not differ between smokers who ceased smoking after the intervention and those who continued smoking. For more details of the process evaluation see Table 3. Smokers in the experimental group reported more often than smokers in the control group that NRT, group counseling and eHealth were discussed. eHealth and group counseling were not mentioned in the control condition at all (Table 3).

**Table 3:** Process evaluation among (ex-) smokers (use and appreciation of the materials and intervention effects on the discussion and use of EBSCIs) measured at 6 months after baseline

***Materials***	***Smokers – experimental condition (N=54) % (n)***				
Poster (paper)	67.3 (37)				
Poster (digital)	22.5 (14)				
Flyers	78.2 (43)				
Placemat	27.3 (15)				
Website during consultation	27.3 (15)				
Appreciation (I found the materials to be….)*	***Smokers – experimental condition Mean (SD)***				
Clear	3.55 (0.8)				
Understandable	3.67 (0.8)				
Educational	3.65 (0.8)				
Score (1–10)	8.00 (1.8)				
***Discussion of materials according to (ex-) smokers***	***Total (N=82) % (n)***	***Experimental condition (N=55) % (n)***	***Control condition (N=27) % (n)***	***χ^2^***	***p***
Number of EBSCIs discussed, mean (SD)	2.44 (1.5)	2.64 (1.7)	2.04 (0.9)	1.836^§^	0.096
Counseling: GP-setting	54.9 (45)	58.2 (32)	48.1 (13)	0.736	0.391
Counseling: coach	23.2 (19)	20.0 (11)	29.6 (8)	0.943	0.331
EHealth	12.2 (10)	18.2 (10)	0 (0)	5.591	**0.018**
Group counseling	7.3 (6)	10.9 (6)	0 (0)	3.178	**0.075**
Telephone counseling	39.0 (32)	40.0 (22)	37.0 (10)	0.067	0.796
NRT	42.0 (35)	54.5 (30)	18.5 (5)	9.608	**0.002**
Pharmacotherapy	58.5 (48)	56.4 (31)	63.0 (17)	0.325	0.569
Other non-EBSCI	6.1 (5)	5.5 (3)	7.4 (2)	0.121	0.728

* 1=strongly disagree; 5=strongly agree.

§ t-test.

### Part 3: Effect evaluation

Table 4 summarizes baseline characteristics and follow-up at 6 months of smokers from both conditions. Smokers from both conditions did not differ on any of the measures at baseline or at follow-up at 6 months, including their use of EBSCIs to support their smoking cessation attempt. Dropout analysis did not find significant differences between smokers followed up and smokers lost to follow-up at 6 months.

**Table 4:** Characteristics of smokers, recruited from May 2019 to May 2020, at baseline and at follow-up at 6 months (N=157)

	*Overall (N=157) n (%)*	*Experimental condition (N=105) n (%)*	*Control condition (N=52) n (%)*	*χ^2^*	*t-test*	*p*
**Baseline**						
**Age** (years), mean (SD)	49.2 (13.6)	49.0 (13.6)	49.6 (13.6)		-0.23	0.819
**Gender** (Female)	77 (49)	51 (48.6)	26 (50.0)	0.03		0.866
**Education level**				0.55		0.760
High	27 (17.2)	17 (16.2)	10 (19.2)			
Medium	39 (24.8)	25 (23.8)	14 (26.9)			
Low	91 (58.0)	63 (60.0)	28 (53.8)			
**Dutch nationality**	154 (98.1)	103 (98.1)	51(98.1)	3.01		0.222
Health status*						
Pulmonary emphysema and/or chronic bronchitis (COPD)	37 (23.6)	23 (21.9)	14 (26.9)	0.47		0.486
Cancer	10 (6.4)	6 (5.7)	4 (7.7)	0.23		0.633
Type 2 diabetes	14 (8.9)	10 (9.5)	4 (7.7)	0.14		0.705
Heart and/or vascular diseases	26 (16.6)	17 (16.2)	9 (17.3)	0.03		0.859
Asthma	25 (15.9)	16 (15.2)	9 (17.3)	0.11		0.739
Depression or major depressive disorder	33 (21.0)	23 (21.9)	10 (19.2)	0.15		0.699
No health conditions	70 (44.6)	49 (46.7)	21 (40.4)	0.56		0.456
Cigarettes smoked/day, mean (SD)	17.6 (8.2)	18.1 (8.4)	16.4 (7.8)		1.25	0.212
**Use of e-cigarettes**				5.421		0.066
No	140 (89.2)	90 (85.7)	50 (96.2)			
Yes, without nicotine	2 (1.3)	1 (1.0)	1 (1.9)			
Yes, with nicotine	15 (9.6)	14 (13.3)	1 (1.9)			
FTND§ score, mean (SD)	6.0 (1.9)	6.1 (2.0)	5.7 (2.0)		0.76	0.448
No previous quit attempts	97 (61.8)	62 (59.0)	35 (67.3)	1.68		0.641
**Readiness to quit in:** (months)				2.82		0.589
<1	105 (66.9)	71 (67.6)	34 (65.4)			
1–3	32 (20.4)	23 (21.9)	13 (25.0)			
4–6	14 (8.9)	10 (9.5)	4 (7.7)			
6–12	1 (0.6)	0 (0.0)	1 (1.9)			
>12	1 (0.6)	1 (1.0)	0 (0.0)			
	***Overall (N=82) % (n)***	***Experimental condition (N=55) % (n)***	***Control condition (N=27) % (n)***	***χ^2^***	***t-test***	***p***
**Follow-up at 6 months**						
**Usage of materials**						
Number of EBSCIs used, mean (SD)	2.29 (1.6)	2.09 (1.4)	2.48 (1.8)			0.270
Counseling: GP-setting	37.8 (31)	63.8 (30)	36.2 (17)	0.52		0.469
Counseling: coach	18.3 (15)	14.5 (8)	25.9 (7)	1.57		0.210
EHealth	8.5 (7)	12.7 (7)	0 (0)	3.76		0.053
Group counseling	11.0 (9)	7.3 (4)	18.5 (5)	4.59		0.101
Telephone counseling	1.2 (1)	1.8 (1)	0 (0)	0.49		0.481
NRT	35.4 (29)	34.5 (19)	37.0 (10)	0.05		0.824
Pharmacotherapy	11.0 (49)	54.5 (30)	70.4 (19)	1.87		0.170
Other non-EBSCI	15.9 (13)	20.0 (11)	7.4 (2)	2.15		0.142
Decisional Conflict Scale, mean (SD)	27.3 (16.1)	28.7 (13.1)	26.0 (19.1)		0.73	0.465

* Combinations of several conditions possible.

§ Fagerström test for nicotine dependence score (range: 1–10).

*Effect on abstinence and smoking behavior*

As a large portion (48%) of data at the measurement at 6 months was missing, resulting in a disproportionate distribution of a low number of participants in both conditions, multiple imputations or multi-level analyses could not be performed on the data set^28^. We therefore report both complete cases and single imputation based on a negative scenario^27^ (Table 5). The group of smokers who indicated to have not smoked a cigarette in the last 24 hours (24-hour point prevalence abstinence) was identical to the group of smokers who reported not having smoked a cigarette in the last 7 days (7-day point prevalence abstinence), and therefore not presented separately in Table 5. We found no significant differences between the two conditions in either scenario for 7-day point prevalence abstinence and 6-month prolonged abstinence.

**Table 5:** Effects on abstinence and smoking behavior per condition

*Characteristics*	*Total % (n)*	*Experimental % (n)*	*Control % (n)*	*χ^2^*	*p*
Complete cases*					
7-day point prevalence abstinence	54.3 (44)	52.7 (29)	57.7 (15)	0.175	0.675
6-month prolonged abstinence	18.5 (15)	34.8 (8)	46.7 (7)	0.537	0.464
**Negative scenario^§^**					
7-day point prevalence abstinence	28.0 (44)	27.6 (29)	28.8 (15)	0.026	0.872
6-month prolonged abstinence	9.6 (15)	7.6 (8)	13.5 (7)	1.374	0.241

* Based on n=82.

§ Based on n=157.

## DISCUSSION

### Part 1: Recruitment and retention

Of the 1663 approached PNs, only a small percentage (4.4%) were willing to participate in the study. Previous studies show such a low percentage is not uncommon for research within the PCS^6,29,30^.

As the PNs in the study recruited a small number of smokers, the recruitment period had to be extended, making the initial 12-month measurement unfeasible. It was, therefore deleted. As suggested by others^31^, we tried to stimulate early recruitment success through postcards with motivational messages, a newsletter (read by 40% of the participating PNs), and telephone calls. The inclusion of financial rewards did not seem to improve recruitment either.

Besides recruitment of smokers, retention rates are also important in RCTs. We had a retention rate of 44.9% (n=157) at baseline and 47.8% (n=82) at 6 months. These retention rates are comparable with other studies with little direct patient–researcher contact^6,31,32^, despite the use of multiple drop-out prevention strategies used, such as sending several reminders for each follow-up questionnaire, promising respondents a €10 voucher for completing all follow-up questionnaires and using abbreviated follow-up questionnaires only assessing three questions regarding smoking behavior to non-responders. Unfortunately, due to privacy reasons, we could not determine the reasons for drop-out.

### Part 2: Process evaluation

Participating PNs reported a higher percentage of usage of all materials than patients, except for the flyer. Use of placemats and website varied between PN and smoker groups, with smokers reporting lower usage percentages during consultations. PNs found the materials clearer and more understandable than smokers. Both groups gave the highest scores for appreciation of materials’ understandability. Differences between PN and smoker groups may be explained by the characteristics of the PNs in the sample, as they were more motivated and had more knowledge of EBSCIs due to their job responsibilities. PNs reported discussing EBSCIs more frequently than smokers, particularly eHealth and group counseling in the control sample. Similar reasons for this discrepancy may be prevalent. Smokers in the experimental condition discussed NRT more often than those in the control condition. This can be regarded as a positive outcome, as NRT is the preferred first option according to Dutch guidelines^13^.

### Part 3: Effect evaluation

We did not find different effects between the experimental and control condition on smoking cessation and actual usage of EBSCIs after referral. Although smokers during the experimental condition were introduced to a wide variety of EBSCIs, their scores on the decisional conflict scale did not differ significantly from the smokers in the control condition. As most EBSCIs do not differ much on aspects such as risks or losses, this may explain the lack of conflict between both groups. Another study in a similar sample suggested that smokers may have already made their choice for an EBSCI before addressing smoking cessation with their PN, based on experiences from their environment, their own previous experiences, and the media^33^.

### Strengths and limitations

This study had several strengths, including proactive outlining of effective smoking cessation methods to both PNs and smokers, addressing the low consensus on EBSCIs among HCPs, and focusing on the effectiveness of the referral aid, materials’ appreciation, and recruitment process. Furthermore, about half (58%) of the smokers with low education were included, a group often difficult to reach^34^, and achieved a high cessation rate of over 50%. Other studies have faced similar recruitment barriers within or via the PCS^29,35^.

Our study has limitations. The limited study sample resulted in an inability to perform multilevel analyses or other statistical analysis while assuring a high statistical validity and possibly preventing type III errors (i.e. correctly rejecting the null hypothesis but for the wrong reasons, for example, when the intervention was not properly implemented). We therefore chose to consider a more descriptive approach to investigate our data, in contrast to the approach described in the protocol publication^23^. Another way to prevent a type III error from occurring, other than including a larger sample, is to monitor more strictly how the intervention is implemented by the HCP. This can be done through self-reporting by PNs or by observation by a trained researcher. However, valid self-reporting requires a lot of time and effort of the PN and might evoke socially desirable answers, producing a distorted picture. Observation by a trained researcher was not possible because of the COVID-19 pandemic and the associated distancing measures. To provide further insights, we recognize the importance of offering additional information on the frequency with which PNs discussed EBSCIs and the percentage of patients involved in these discussions. A comparative analysis of patient responses would enhance the understanding of the intervention’s impact.

Second, our PNs participating may have been a select group who are more open to innovations or are more interested in smoking cessation-related healthcare (selection bias). A consequence might be that the results could be even less positive in a broader population. As PNs often report non-adherence to the Dutch Cessation Guidelines because of time or cost constraints^15^, another explanation for the low participation rate might be that PNs are discouraged by the burden of the additional research elements associated with RCTs. Reflecting on the reasons provided, it is imperative for future research to delve into these reasons comprehensively. Understanding the factors influencing PN participation is crucial for refining recruitment strategies. We acknowledge that out of the 1600 PNs approached, some may not have even read the invitation due to the high volume of research requests they receive. Exploring more personalized recruitment strategies is essential for future investigations.

Finally, the planned cost-effectiveness analysis (CEA) and cost-utility analysis (CUA)^23^ were not executed because of the small sample size and lack of behavioral results (mainly, no differences in quitting behavior between both groups).

### Recommendation for practice

In light of the issues described, we would like to propose three recommendations for practice: 1) recruitment within an RCT or other research study, 2) providing smoking cessation counseling and referral to EBSCIs within the PCS, and 3) providing smoking cessation counseling and referral to EBSCIs within the PCS outside the PCS.

First, our findings suggest that PNs find it difficult to recruit smokers for an RCT, possibly due to time constraints (heavy workload) or different priorities. This implies that alternative approaches should be considered, such as engaging specifically trained and compensated personnel to assist in patient recruitment in coordination with the PN and smokers.

Second, when looking at the situation within the PCS, time or cost constraints often play a large role in the adherence of PNs to the smoking cessation guidelines, including referral to EBSCIs^15^. Currently, referring smokers to cessation methods outside Dutch practices may imply that the PN/PCS will not receive the patient-related smoking cessation reimbursement from the patient’s healthcare insurance, as these methods may not be covered by the patients’ policies. Hence, exploring the feasibility of incorporating smoking cessation coaching outside the general practice into healthcare insurance could help alleviate the burden on the PNs, including the coaching of smokers to be included in the reimbursement system.

Third, it may be important to explore whether there are additional venues outside the PCS to talk about smoking cessation to divide forces, reach (dividing the responsibility) and persuade more smokers to quit (by spreading the message through multiple sources), for example, through other HCPs such as dentists, midwives or social workers, or through community health workers and the social domain.

Although research has found other HCPs also encounter barriers such as lack of time and training^10^, spreading the workload can help lower the total individual pressure. To achieve this, appropriate educational options, possibly a simplified version of the 5As or Dutch guidelines such as the ask-advise-refer (AAR) strategy, have already been tried out or proven effective in other settings^9^. Furthermore, HCPs should be able to claim reimbursement for these actions requiring additional adjustment to the current funding system of the Dutch Health care^36^. Other entryways for reaching smokers need further attention as well, such as the workplace^6^, the Internet or directly to known smoking households in the social domain^5^.

## CONCLUSIONS

This study aimed to explore the use and effect of referral aid from the perspectives of PNs and smokers by investigating the course of recruitment and conducting a process and effect evaluation. Recruitment of both PNs and smokers resulted in low levels of participation. Overall, PNs found the materials clear and understandable. Smokers had similar but (slightly) less-positive opinions. However, the referral aid was marginally used, and the groups of smokers and smokers who quit did not marginally differ on discussion and use of EBSCIs, nor differed on abstinence. As the main finding concerned a low level of participation and use of the referral aid by PNs, further research should aim to assess how to better involve PNs and smokers when recruiting for an RCT and how to foster effective counselling. Additional research should also look deeper into barriers to referral of both PNs and smokers and how to best stimulate referral to EBSCIs and help smokers make a decision, for example, by implementing a simplified strategy such as the AAR, both within the PCS and outside the PCS, by involving other HCPs and options outside healthcare such as the workplace and the social domain.

## Data Availability

The data supporting this research are available from the authors on reasonable request.
